# Cerebrospinal fluid sCD27 levels indicate active T cell-mediated inflammation in premanifest Huntington's disease

**DOI:** 10.1371/journal.pone.0193492

**Published:** 2018-02-23

**Authors:** Valter Niemelä, Joachim Burman, Kaj Blennow, Henrik Zetterberg, Anders Larsson, Jimmy Sundblom

**Affiliations:** 1 Department of Neuroscience, Neurology, Uppsala University, Uppsala, Sweden; 2 Department of Psychiatry and Neurochemistry, the Sahlgrenska Academy at the University of Gothenburg, Mölndal, Sweden; 3 Clinical Neurochemistry Laboratory, Sahlgrenska University Hospital, Mölndal, Sweden; 4 Department of Molecular Neuroscience, UCL Institute of Neurology, Queen Square, London, United Kingdom; 5 UK Dementia Research Institute at UCL, London, United Kingdom; 6 Department of Medical Sciences, Clinical Chemistry, Uppsala University, Uppsala, Sweden; 7 Department of Neuroscience, Neurosurgery, Uppsala University, Uppsala, Sweden; Institut d'Investigacions Biomediques de Barcelona, SPAIN

## Abstract

**Introduction:**

Huntington’s disease (HD) is a neurodegenerative disorder, but evidence also suggests neuroinflammation in the pathogenesis. The immune mechanisms involved and the timing of their activation need further clarification.

**Methods:**

A clinically well-characterized HD cohort and gene negative controls were enrolled. YKL-40 reflecting innate immunity and sCD27, a marker of adaptive immunity, were measured across disease stages. Comparisons were made with markers of neurodegeneration: neurofilament light (NFL), total-tau (T-tau), and phospho-tau (P-tau).

**Results:**

52 cross-sectional cerebrospinal fluid samples and 23 follow-up samples were analyzed. sCD27 was elevated in manifest HD and premanifest gene expansion carriers, whereas controls mostly had undetectable levels. YKL-40 showed a trend toward increase in manifest HD. sCD27 correlated with YKL-40 which in turn was closely associated to all included markers of neurodegeneration. YKL-40, NFL, and both forms of tau could all independently predict HD symptoms, but only NFL levels differed between groups after age-adjustment.

**Conclusion:**

Increased sCD27 in premanifest HD is a sign of T cell-mediated neuroinflammation. This finding is novel since other reports almost exclusively have found early involvement of innate immunity. Validation of sCD27 in a larger HD cohort is needed. The role of adaptive immunity in HD needs further clarification, as it may hasten disease progression.

## Introduction

Huntington’s disease (HD) is an autosomal dominant neurodegenerative disorder caused by an expansion of the polyglutamine tract on exon 1 in the HTT-gene [1]. Symptoms are progressive and include motor impairment, psychiatric symptoms [2] and cognitive decline [3]. There are no registered disease-modifying therapies, but promising clinical trials are now targeting mutant huntingtin expression, and neuroinflammation [4].

Although HD is a neurodegenerative disorder, immune system dysfunction and inflammation, is part of the pathogenesis [5–7]. While astrocytes and microglia have a function in normal CNS tissue repair, their activation is suspected to contribute to neurodegeneration in HD [8]. Monocytes, macrophages and microglia that express mutant huntingtin are hyperactive when stimulated by lipopolysaccharide, secreting more of interleukins (IL) 6 and -8 than normal cells. Identical elevation of IL-6- and IL-8, which are involved in activation of the NF-kB pathway, has been found both in blood and cerebrospinal fluid (CSF) of HD patients [5]. Microglial activation years before motor onset is also supported by PET imaging studies [6, 9]. These studies suggest that innate immune dysfunction is an early disease mechanism, while adaptive immunity is hypothesized be a late and secondary feature.

While biofluid biomarkers for disease progression are not generally used in the clinical setting today, there is a need for objective biomarkers to empower clinical trials, avoiding placebo effects and limitations of clinical assessment. When treatments are available, these markers could also guide the timing for treatment initiation and evaluation of effect. CSF is a good source for detection of biomarkers in HD [10]. Cross-sectional studies agree that the axonal damage marker CSF neurofilament light (NFL) is a promising HD biomarker [11–14]. T-tau, reflecting damage of neurons in proximity to the soma, has also been proposed as a possible biomarker [11, 15, 16]. CSF Phosphorylated tau (P-tau), which is associated to formation of neurofibrillary tangles in neurodegenerative disorders [17], has not been studied in HD.

CSF studies of inflammatory markers from human HD gene expansion carriers remain rare. CSF YKL-40 (chitinase 3-like protein, CHI3L1) is secreted by astrocytes, and is increased in many inflammatory CNS disorders [18]. While the exact function of YKL-40 is unknown, it is hypothesized to be an agent of the innate immune system, involved in tissue remodeling during inflammation [19]. Elevated YKL-40 concentrations have been reported in HD [14, 20].

Soluble CD27 receptor (sCD27) in CSF is a highly specific marker of intra-thecal T-cell mediated inflammation [21], and has not been studied in HD before.

Longitudinal CSF studies in HD are still lacking [10]. Following an HD cohort and performing repeated samples could provide knowledge about how molecular changes and disease development are linked. Longitudinal results on blood NFL were recently published [22], but longitudinal studies of CSF NFL are still needed, since it is already being included as a pharmacodynamic biomarker in clinical drug trials.

In the present work, we analyzed YKL-40 and sCD27 in CSF across HD stages in an exploratory analysis to detect any elevation before motor onset. By including NFL and tau-proteins, the association between inflammation and axonal- as well as neuronal damage could be studied. With repeated samples, we also aimed to find temporal associations between the markers and the events that they represent.

## Materials and methods

### Definition of participants and clinical assessment

All participants were recruited from the neurology outpatient clinic at Uppsala university hospital, Uppsala, Sweden. Manifest HD patients, premanifest gene expansion carriers and gene negative controls were asked to participate. Manifest HD was defined as CAG repeat length > 35 and diagnostic confidence level (DCL) of 4. Premanifest gene expansion carriers were defined as CAG repeat length > 35 and DCL below 4 [23]. Controls who had a family history of HD had CAG repeat length < 35. Controls who had no family history of HD were not genetically tested. Clinical assessment included total motor score [24], stroop interference, and total functional capacity [25].

Disease burden [26], a function of CAG repeat length and age was calculated ((CAG_n_ -35.5) x age) for all gene expansion carriers, as well as the 5-year probability of onset [27] for the premanifest gene expansion carriers

The study was approved by the Regional Ethical Review Board in Uppsala (DNR 2012/274). All participants provided written informed consent.

### CSF sample collection and handling

CSF sample collection was standardized for procedure, materials and handling, but the timing of lumbar puncture and relation to meals varied. Polypropylene tubes and collecting vessels were used to avoid protein adsorption. The CSF was put on ice and centrifuged at 4 degrees Celsius and 1300 G for 10 minutes. The supernatant was pipetted off for storage at minus 70 degrees Celsius until the time of analysis. Median storage time for the baseline samples was 2.1 years for the premanifest gene expansion carriers (range 0.9–4.6 years), 2.4 years for the manifest HD group (range 0.2–4.6 years) and 2.2 years for the controls (range 1.2–4.2 years).

### Biochemical analyses

The protein analyses were performed at the Clinical Neurochemistry Laboratory, Sahlgrenska University Hospital, Mölndal campus, Sweden, except sCD27 which was analyzed at the dept. of clinical chemistry, Uppsala University Hospital, Uppsala, Sweden. The analyses were performed by board-certified laboratory technicians blinded to clinical information. All samples were run in a single batch, using the same reagents, to reduce inter-assay variation.

CSF T-tau was measured using a sandwich enzyme-linked immunosorbent assay (ELISA; INNOTEST hTAU-Ag, Fujirebio Europe, Gent, Belgium) designed to measure all tau isoforms irrespective of phosphorylation status. Phosphorylated tau (P-tau181) was measured using a sandwich ELISA (INNOTEST phosphotau[181P], Fujirebio Europe, Gent, Belgium). CSF YKL-40 levels were analyzed using a commercial ELISA kit (R&D Systems, Minneapolis, MN, USA), while CSF NF-L levels were measured by the NF-light^®^ ELISA kit (UmanDiagnostics AB, Umea, Sweden), both according to the instructions by the manufacturers.

Soluble human CD27/TNFRSF7 was analyzed by a commercial sandwich ELISA kit, (Human CD27/TNFRSF7 DY382, R&D Systems, Minneapolis, MN, USA), according to the instructions by the manufacturer. The limit of detection for the assay was 40 pg/mL and the highest standard point was 8000 pg/mL. The intra-assay coefficient of variation (CV) for the assay was 4.5% and the total CV was approximately 6%.

### Statistical analysis

Intergroup differences in protein levels were tested with nonparametric tests (Mann-Whitney U test). For all proteins that showed an association to age or sex, this variable was included as a covariant in a linear regression model. Correlations were calculated on all gene expansion carriers pooled into one group, using Spearman rank correlation. All statistical analyses were calculated on the cross-sectional sample results. Analysis of longitudinal dynamics was qualitative and involved no statistical tests. The level of significance was defined at p < 0.05. Statistical analyses were performed using SPSS version 24, and graphs were created in GraphPad Prism version 7.

## Results

The study enrolled 52 individuals with cross-sectional CSF samples. Of these 14 were manifest HD patients, 13 were premanifest gene expansion carriers and 25 were gene negative controls. 11 manifest HD patients, 9 premanifest gene expansion carriers and 3 controls donated follow-up samples after 1–4 years (Table 1). During the study one premanifest gene expansion carriers converted to manifest HD.

**Table 1:** Characteristics of study population.

Category	n =	Longitudinal sample	Mean age (range)	Female (%)	Mean CAG_n_ (range)	Mean Disease burden (range)	MeanTFC (range)
**Manifest HD**	14	11	51 (30–72)	5 (36)	43.5 (39–49)	382 (252–493)	9.6 (3–13)
**Premanifest HD**	13	9	36 (19–56)	6 (46)	43.6 (40–54)	265.3 (149–371)	13 (0.0)
**Controls**	25	3	24 (18–37)	12 (48)	N/A	N/A	N/A

HD, Huntington’s disease; Premanifest HD; premanifest gene expansion carriers; CAG_n_, CAG expansion length; TFC, Total Functional Capacity; Disease burden, (CAG_n_-35.5) x age.

### Associations of proteins with age and sex

In the pooled HD groups YKL-40 correlated with age (r = 0.66 p = 0.0002), as did NFL (r = 0.56 p = 0.002), and both forms of tau (T-tau r = 0.56 p = 0.002; P-tau r = 0.55 p = 0.003). Although no significant correlations were found between age and any of the markers in the controls, coefficients of T-tau (r = 0.11 p = 0.6) and P-tau (r = 0.06 p = 0.8) were of very low magnitude compared to NFL (r = 0.38 p = 0.06) and YKL-40 (r = 0.27 p = 0.2). The effect of age on sCD27 has been investigated at our site in an unpublished study where there was no association (S1 Table including normal values for sCD27). Therefore, adjustment for age was not performed. Sex was significantly associated to sCD27 in the pooled HD groups (p = 0.011) where males had 2.2 times higher sCD27 concentrations (mean 235.9 pg/ml, SD 111.5) than females (mean 106.8 pg/ml, SD 94.1). No other proteins were associated to sex.

### Adjusted protein concentration differences between groups

**Fig 1:**
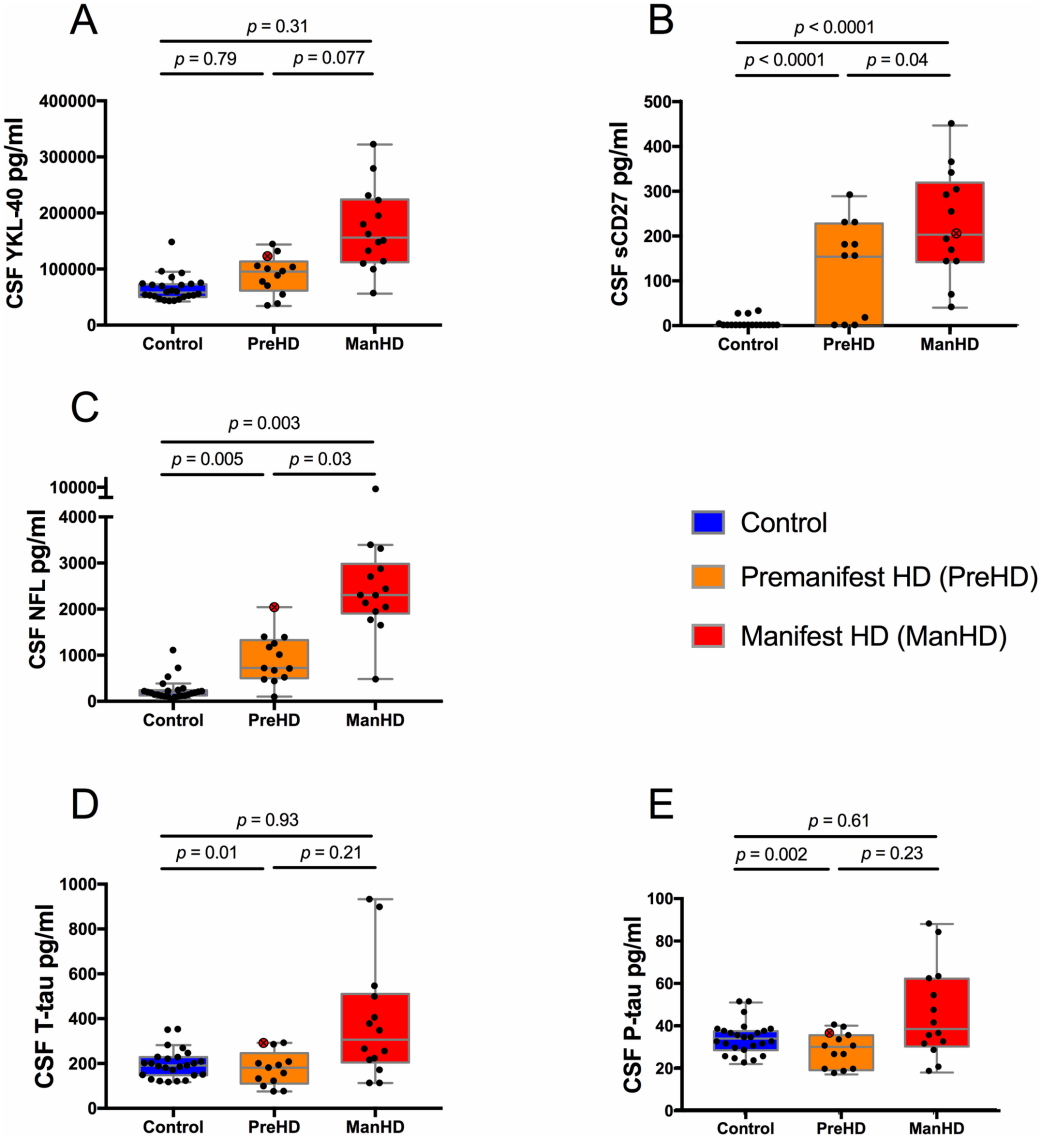
Concentrations of YKL-40, sCD27, NFL, T-tau and P-tau by group. Protein concentrations plotted un-adjusted by group. p-values are adjusted for age or sex, as described below. Boxes show first and third quartiles, the central bands show the median, and the whiskers show data within 1.5 IQR of the median. Red point ID indicates a subject who converted from premanifest to manifest disease during the study. (**A**) YKL-40 levels differed between all groups before, but not after adjustment for age. (**B**) Sex-adjusted sCD27 concentrations were higher in manifest HD than premanifest gene expansion carriers, and close to zero in controls. (The converter had sCD27 analyzed only on the 2^nd^ sample). (**C**) Age-adjusted NFL concentrations differed between all groups. Levels of (**D**) T-tau and (**E**) P-tau were higher in manifest patients compared to premanifest before adjustment for age, but not after. The premanifest group had lower levels than controls, but the difference was significant only after adjustment for age.

Fig 1 shows the concentrations of all markers in the three groups. YKL-40 levels (Fig 1A) did not differ between groups after adjustment for age, although a trend toward higher concentrations was noted in the manifest group compared to the premanifest gene expansion carriers.

sCD27 concentrations (Fig 1B) were clearly elevated in premanifest gene expansion carriers (n = 11), compared with controls (n = 18) (p = 0.002, adjusted for sex p < 0.0001). The manifest HD group (n = 13) had non-significantly higher sCD27 concentrations, compared with premanifest gene expansion carriers (n = 11) (p = 0.072). After adjustment for sex, however, the difference was significant (p = 0.038).

Age-adjusted NFL levels (Fig 1C) were higher in premanifest gene expansion carriers compared with controls. Manifest HD patients had higher age-adjusted levels of NFL than premanifest gene expansion carriers.

Regarding both forms of tau (Fig 1D and 1E), the premanifest gene expansion carriers had a non-significant trend toward lower levels compared with controls (T-tau p = 0.45; P-tau p = 0.11), but after adjustment for age the difference was significant. Manifest HD patients had higher levels than premanifest gene expansion carriers before adjustment for age (T-tau p = 0.012; P-tau p = 0.021), but not after (T-tau p = 0.21; P-tau p = 0.23).

### Association with disease progression

All of the included markers, except sCD27 correlated with disease burden (YKL-40 r = 0.71 p < 0.0001; sCD27 r = 0.28 p = 0.19; NFL r = 0.80 p < 0.0001; T-tau r = 0.62 p = 0.0006; P-tau r = 0.56 p = 0.002). YKL-40 and NFL also correlated significantly with 5-year risk of onset in the premanifest gene expansion carriers (YKL-40 r = 0.77 p = 0.002; NFL r = 0.69 p = 0.009) whereas sCD27, T-tau and P-tau did not (sCD27 r = 0.57 p = 0.056; T-tau r = 0.53 p = 0.061; P-tau r = 0.43 p = 0.14).

### Correlation with clinical test scores

Correlations of markers with total motor score (TMS), stroop interference and total functional capacity (TFC) are presented in full in Table 2. In summary, YKL-40 correlated with TMS and stroop interference, after adjustment for disease burden.

**Table 2:** Correlations with clinical test scores for all markers.

		Disease burden- adjusted *p*-value	Age-adjusted *p*-value	*p*-value	Unadjusted correlation
**YKL-40**	**TMS**	0.028	0.018	0.0001	0.67
	**Stroop interference**	0.002	0.006	0.0003	-0.72
	**TFC**	0.13	0.14	0.001	-0.59
					
**sCD27**	**TMS**	0.52	N/A	0.21	0.27
	**Stroop interference**	0.25	N/A	0.078	-0.38
	**TFC**	0.12	N/A	0.66	-0.10
					
**NFL**	**TMS**	0.006	0.0001	< 0.0001	0.83
	**Stroop interference**	0.069	0.001	< 0.0001	-0.83
	**TFC**	0.003	0.0003	0.0002	-0.67
					
**T-tau**	**TMS**	0.044	0.033	0.001	0.60
	**Stroop interference**	0.096	0.12	0.001	-0.61
	**TFC**	0.24	0.23	0.007	-0.50
					
**P-tau**	**TMS**	0.037	0.027	0.002	0.57
	**Stroop interference**	0.049	0.074	0.001	-0.60
	**TFC**	0.19	0.18	0.014	-0.47

Abbreviations: NFL, neurofilament light; T-tau, Total-tau; P-tau, Phosphorylated tau; sCD27, soluble CD27 receptor; YKL-40 / Chitinase 3 Like-1 (CHI3L1); TMS, Total Motor Score; TFC, Total Functional Capacity. Disease burden = (CAG_n_-35.5) x age.

sCD27 did not correlate significantly with any of the clinical scores.

NFL correlated with TMS and TFC after adjustment for disease burden.

Both forms of tau correlated with TMS after adjustment for disease burden, but their correlations with stroop interference and TFC were lost after adjustment for age.

### Intercorrelation between markers

Correlations are listed in Table 3. The strongest correlation observed was between T-tau and P-tau, where one could explain most of the variation in the other (r^2^ = 0.96). YKL-40 had strong correlations with all markers of neurodegeneration. sCD27 was correlated to the other marker of inflammation YKL-40, but also to both forms of tau. Strong correlations were observed between all markers of neurodegeneration.

**Table 3:** Intercorrelation between markers in the two HD groups.

	**NFL**	**T-tau**	**P-tau**	**sCD27**
**T-tau**	0.858 ***			
**P-tau**	0.828 ***	0.978 ***		
**sCD27**	0.362	0.531 **	0.510 *	
**YKL-40**	0.843 ***	0.869 ***	0.814 ***	0.532 **

Abbreviations: NFL, neurofilament light protein; T-tau, Total-tau protein; P-tau, Phosphorylated tau protein; sCD27, soluble CD27 receptor; YKL-40, Chitinase 3-Like 1 protein, (CHI3L1). All correlations are derived from baseline cross-sectional samples of all gene expansion carriers.

*(p < 0.05),

**(p <0.01),

***(p <1x10^-6^).

### Longitudinal protein dynamics

**Fig 2:**
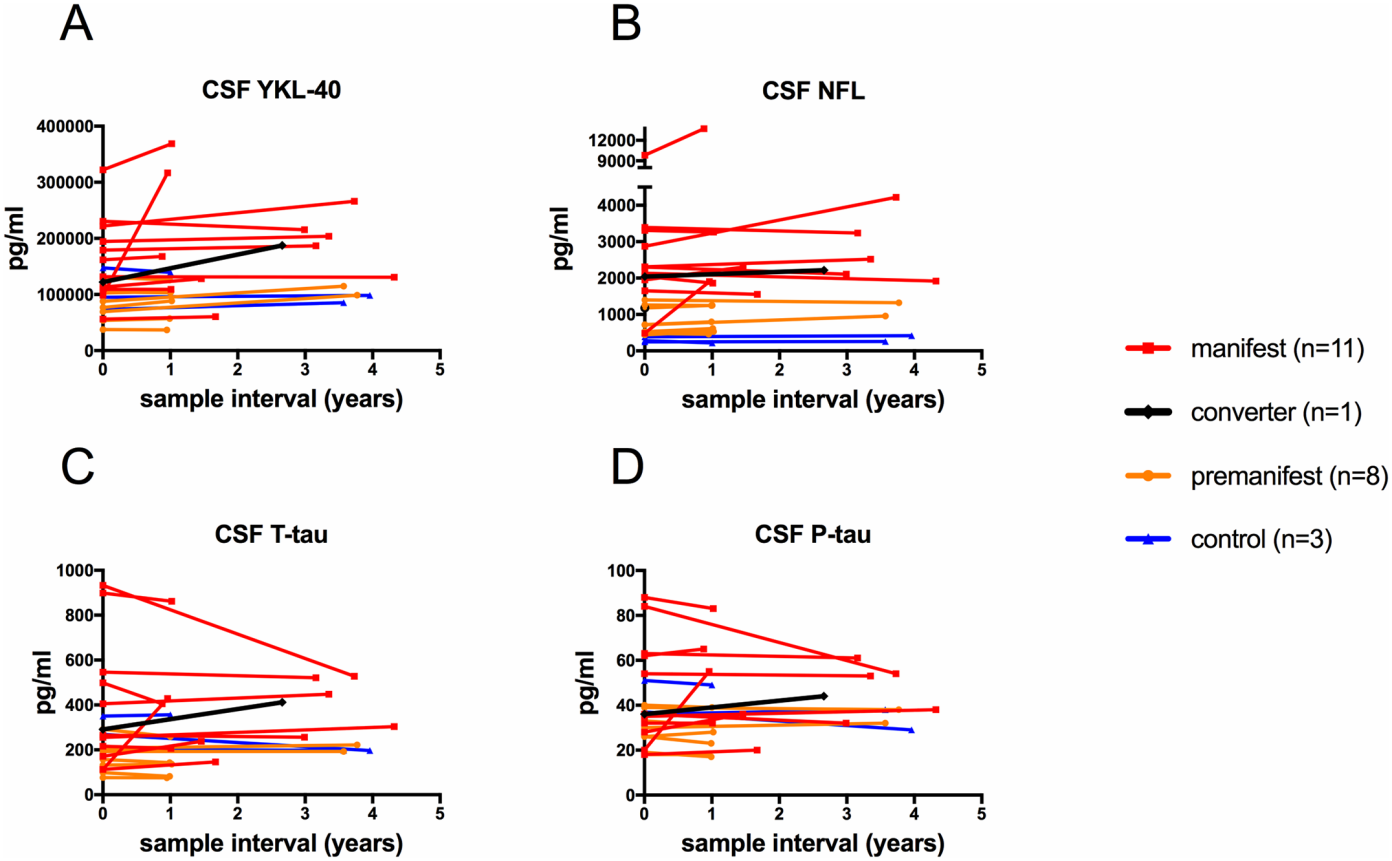
Longitudinal dynamics of biomarkers in Huntington’s disease including YKL-40, NFL, T-tau and P-tau. Longitudinal biomarker dynamics of (**A**) Neurofilament light levels (NFL), (**B**) YKL-40 and (**C**) Total Tau (T-tau), and (**D**) Phospho-tau (P-tau). Each line represents one individual, starting at the first CSF sample and protein concentration, finishing at the time of the second sample and concentration.

Fig 2 presents longitudinal dynamics of all markers except sCD27, of which no repeated samples were available. The mean sample interval in years per group was as follows; premanifest gene expansion carriers 1.77 (range 0.95–3.77), manifest HD 2.23 (range 0.88–4.32), controls 2.87 (range 1.07–3.96).

One subject was defined as premanifest at the time of the first visit and converted to manifest disease before the second CSF sample was drawn. This subject had the highest NFL value in the premanifest group (Fig 2B), situated in the range of the manifest HD group. The same subjects second sample showed slowly rising NFL and increasing levels of YKL-40 (Fig 2A), T-tau (Fig 2C) and P-tau (Fig 2D).

P-tau and T-tau showed an almost identical pattern over time. (Fig 2C and 2D).

## Discussion

In this exploratory CSF study, by analyzing sCD27 in HD, we report evidence for T cell-mediated neuroinflammation in premanifest gene expansion carriers. YKL-40 levels were similar in the premanifest group compared to controls, showing a non-significant trend toward elevated levels in manifest HD. Despite the intergroup differences, sCD27 concentrations did not correlate with clinical test scores and correlations with the included markers of neurodegeneration were weak. Due to low mean sCD27 concentrations, around the assay’s limit of detection, the differences in concentrations could not be quantified in a purely linear fashion. Therefore, we can neither confirm nor exclude, the possibility of at least weaker correlations with HD symptoms. In contrast to sCD27, YKL-40 independently predicted HD symptoms, and was closely associated to all markers of neurodegeneration. Vinther-Jensen et al. described YKL-40 increase as a late feature in HD [14, 28], while Rodrigues et al. found elevated YKL-40 in a small group of premanifest gene expansion carriers [20]. The latter finding remains to be replicated. This late increase is in line with post-mortem studies, that found astrocytosis in moderate, but not the earliest pathological grades of HD in humans [29], and the HD mouse model R6/2, where Glial fibrillary acid protein (GFAP) was elevated in astrocytes of moderate/late disease stages as a sign of classical astrocyte activation [30]. YKL-40 secretion may be triggered by already activated microglia and cytokines of the NF-kB pathway [19].

To the best of our knowledge, this is the first CSF study that directly implicates an adaptive immune response before motor onset in HD. However, mutant huntingtin expression in peripheral T cells has previously been linked to disease progression, and this may in part explain T cell involvement by a cell-autonomous immune mechanism [7]. Our finding opens up questions about how T cells might contribute to pathology in HD. For instance, which antigens are recognized by the activated T cells? To find answers, revisiting animal models, and immune cell studies in-vitro may be needed.

This study also confirms the ability of NFL to independently predict the clinical HD phenotype, beyond age and CAG repeats. In the longitudinal profile of NFL, differences between manifest subjects, premanifest gene expansion carriers and controls appeared to be more stable over time, compared with the other markers. One subject converted to manifest disease during follow-up time between samples. In this case, we note that YKL-40 and both forms of tau rose toward levels similar to other manifest HD subjects at the second sample, whereas baseline NFL was already in the range of the manifest group, in line with the hypothesis of NFL as a suitable HD biomarker, that rises long before motor onset [11, 14].

Limitations in this study include the exploratory nature with a small sample. Different ages between groups was a concern and perhaps some protein level intergroup differences would have remained significant, had this not been the case. The potential effects of antidepressants which were more common in the premanifest group compared with controls, and antipsychotics that were only used by manifest HD subjects were not assessed.

However, we hope that these findings may be of relevance regarding the involvement of adaptive immunity in HD and for those planning clinical trials with NFL as a surrogate endpoint. sCD27 may come to serve as a marker of inflammation in HD. First, however, our findings will need to be validated in a larger HD cohort. New assays may also be needed for optimal sCD27 quantification in the lower range.

## Supporting information

S1 Table Normal values for sCD27. (DOCX)

S2 Table Dataset including clinical characteristics of study participants and protein concentrations. (XLSX)
