# Supplementary material for: Cerebrospinal fluid sCD27 levels indicate active T cell-mediated inflammation in premanifest Huntington's disease
Source: PLoS One. 2018 Feb 23;13(2):e0193492. doi: 10.1371/journal.pone.0193492 (PMC5825143; doi:10.1371/journal.pone.0193492)
Supplement: S1 Table — (DOCX) [file pone.0193492.s001.docx]

**S1 Table.** Normal values for sCD27 were determined in another yet unpublished study of sCD27 in neurological disease. The study subjects were healthy volunteers (n=47); patients who were investigated for suspected neurological disease where a neurological diagnosis was ruled out (n=36); and patients who underwent spinal anaesthesia for minor urological surgery, with no prior history of a neurological disease (n=42). See tabulated data below. A possible age correlation was investigated through a correlation analysis in each group. In none of the groups a significant correlation between sCD27 and age was found (healthy volunteers Spearman r=0.16, p=0.27; investigated without disease Spearman r=-0.08, p=0.66; urologic controls Spearman r=0.04, p=0.80).

|  | **n** | **M/F** | **Median Age (Range)** | **sCD27 Mean Values ±SD (95% CI)** | **sCD27 Median Values (Range)** |
| --- | --- | --- | --- | --- | --- |
| Healthy Volunteers | 47 | 18/29 | 37 (18-74) | 50 ±88 (95% CI 24-76) | 16 (0-428) |
| Investigated Without Disease | 36 | 8/28 | 41 (19-85) | 47 ±61 (95% CI 26-68) | 13 (0-214) |
| Urologic Controls | 42 | 40/2 | 68 (45-85) | 280 ±130 (95% CI 239-321) | 252 (105-727) |
